# Supplementary material for: Lysosome lipid signalling from the periphery to neurons regulates longevity
Source: Nat Cell Biol. 2022 Jun 9;24(6):906–16. doi: 10.1038/s41556-022-00926-8 (PMC9203275; doi:10.1038/s41556-022-00926-8)
Supplement: Supplementary file 1 — Reporting Summary [file 41556_2022_926_MOESM1_ESM.pdf]

nature portfolio

Corresponding author(s): Prof. Meng C. Wang

Last updated by author(s): Mar 23, 2022

Reporting Summary

Nature Portfolio wishes to improve the reproducibility of the work that we publish. This form provides structure for consistency and transparency in reporting. For further information on Nature Portfolio policies, see our Editorial Policies and the Editorial Policy Checklist.

Statistics

For all statistical analyses, confirm that the following items are present in the figure legend, table legend, main text, or Methods section.

n/a	Confirmed	
☐	☒	The exact sample size (*n*) for each experimental group/condition, given as a discrete number and unit of measurement
☐	☒	A statement on whether measurements were taken from distinct samples or whether the same sample was measured repeatedly
☐	☒	The statistical test(s) used AND whether they are one- or two-sided<br>*Only common tests should be described solely by name; describe more complex techniques in the Methods section.*
☒	☐	A description of all covariates tested
☐	☒	A description of any assumptions or corrections, such as tests of normality and adjustment for multiple comparisons
☒	☐	A full description of the statistical parameters including central tendency (e.g. means) or other basic estimates (e.g. regression coefficient) AND variation (e.g. standard deviation) or associated estimates of uncertainty (e.g. confidence intervals)
☐	☒	For null hypothesis testing, the test statistic (e.g. *F*, *t*, *r*) with confidence intervals, effect sizes, degrees of freedom and *P* value noted<br>*Give P values as exact values whenever suitable.*
☒	☐	For Bayesian analysis, information on the choice of priors and Markov chain Monte Carlo settings
☒	☐	For hierarchical and complex designs, identification of the appropriate level for tests and full reporting of outcomes
☒	☐	Estimates of effect sizes (e.g. Cohen's *d*, Pearson's *r*), indicating how they were calculated

Our web collection on statistics for biologists contains articles on many of the points above.

Software and code

Policy information about availability of computer code

Data collection	Fluorescent images were taken using confocal FV3000 (Olympus). Western-blot images were acquired using Image Quant LAS 500. Free fatty acids were identified using lipidsearch 4.2.27 software (Thermo Fisher Scientific).
Data analysis	The commercial softwares used for data collection are specified in the methods section.

For manuscripts utilizing custom algorithms or software that are central to the research but not yet described in published literature, software must be made available to editors and reviewers. We strongly encourage code deposition in a community repository (e.g. GitHub). See the Nature Portfolio guidelines for submitting code & software for further information.

Data

Policy information about availability of data

All manuscripts must include a data availability statement. This statement should provide the following information, where applicable:

- Accession codes, unique identifiers, or web links for publicly available datasets
- A description of any restrictions on data availability
- For clinical datasets or third party data, please ensure that the statement adheres to our policy

All data generated or analyzed during this study are included in this manuscript and the Extended Data Materials. The RNA-seq data has been deposited into the NCBI Sequence Read Archive (SRA) and the accession codes for each biological sample are SAMN25414087, SAMN25414088, SAMN25414089, SAMN25414090, SAMN25414091, SAMN25414002, SAMN25414093, SAMN25414094, SAMN25414095, SAMN25414096, SAMN25414097, SAMN25414098, and the BioProject accession code is PRJNA801907 (https://www.ncbi.nlm.nih.gov/bioproject/PRJNA801907).

Field-specific reporting

Please select the one below that is the best fit for your research. If you are not sure, read the appropriate sections before making your selection.

☒ Life sciences ☐ Behavioural & social sciences ☐ Ecological, evolutionary & environmental sciences

For a reference copy of the document with all sections, see nature.com/documents/nr-reporting-summary-flat.pdf

Life sciences study design

All studies must disclose on these points even when the disclosure is negative.

Sample size	No sample-size calculation was performed. Sample size was used based on previous literature and recognized in the worm field. For each experiment, n values are provided in the figure legends and in the section of Statistics and Reproducibility. The amount of animals used for each experiment is reported in the Method session.
Data exclusions	No data were excluded from the analysis in the study
Replication	All experimental replications were repeated at least three independent times and the detailed information is specified in Figure Legends and Statistics And Reproducibility
Randomization	Worms with the same genotype were randomly chosen and grouped into experiments.
Blinding	Investigators were not blinded, as for each experiment and analysis worms from different genotypes were compared to wild-type/control

Reporting for specific materials, systems and methods

We require information from authors about some types of materials, experimental systems and methods used in many studies. Here, indicate whether each material, system or method listed is relevant to your study. If you are not sure if a list item applies to your research, read the appropriate section before selecting a response.

Materials & experimental systems

n/a	Involved in the study	
☐	☒	Antibodies
☒	☐	Eukaryotic cell lines
☒	☐	Palaeontology and archaeology
☐	☒	Animals and other organisms
☒	☐	Human research participants
☒	☐	Clinical data
☒	☐	Dual use research of concern

Methods

n/a	Involved in the study	
☒	☐	ChIP-seq
☒	☐	Flow cytometry
☒	☐	MRI-based neuroimaging

Antibodies

Antibodies used	Anti-HA, Rabbit, Cell Signaling, #C29F4, https://www.cellsignal.com/products/primary-antibodies/ha-tag-c29f4-rabbit-mab/3724<br>Anti-beta-actin, Mouse, Santa Cruz, sc-47778, https://www.scbt.com/p/beta-actin-antibody-c4
Validation	Validation statement from the company: HA-Tag (C29F4) Rabbit mAb detects exogenously expressed proteins containing the HA epitope tag in all the species specified including C. elegans. In our study, wild type worms that do not express HA-tagged proteins were used as negative controls for the Anti-HA antibody (Cell Signaling, #C29F4). Validation of Anti-beta-actin, Mouse, Santa Cruz, sc-47778 was previously described (PMID:27534274, PMID:32966783, PMID: 30599151).

Animals and other organisms

Policy information about studies involving animals; ARRIVE guidelines recommended for reporting animal research

Laboratory animals	Caenorhabditis elegans used in this study:<br><br>N2 Bristol Strain<br>raxIs3[ges-1p::lipl-4::sl2-GFP; myo-2p::mCherry]<br>egl-21(n476)<br>raxIs3[ges-1p::lipl-4::sl2-GFP; myo-2p::mCherry];egl-21(n476)<br>fat-3(wa22)<br>raxIs3[ges-1p::lipl-4::sl2-GFP; myo-2p::mCherry];fat-3(wa22)<br>fat-1(wa9)

raxIs3[ges-1p::lipl-4::sl2-GFP; myo-2p::mCherry];fat-1(wa9)
lbp-3(rax60)
raxIs3[ges-1p::lipl-4::sl2-GFP; myo-2p::mCherry];lbp-3(rax60)
raxIs141[lbp-3p::lbp-3::sl2-GFP; myo-2p::mCherry]
raxIs119 [lbp-3p::lbp-3::gfp, myo-2p::mCherry]
Ex440[ges-1p::lbp-3::sl2-GFP;myo-2p::mCherry]
Ex441[ges-1p::lbp-3::sl2-GFP;myo-2p::mCherry]
Ex426[ges-1p::lbp-2SL2GFP;myo-2p::mCherry]
Ex545[ges-1p::nslbp3 Tg::sl2-GFP; myo-2p::mCherry]
Ex546[ges-1p::nslbp3 Tg::sl2-GFP; myo-2p::mCherry]
raxIs22[nlp-11Tg::sl2-GFP;myo-2p::mCherry]
nlp-11(rax51)
raxIs3[ges-1p::lipl-4::sl2-GFP; myo-2p::mCherry];nlp-11(rax51)
raxEx70[ges-1p::nlp-11::sl2-GFP; myo-2p::mCherry]
raxEx71[ges-1p::nlp-11::sl2-GFP; myo-2p::mCherry]
raxEx72[rab-3p::nlp-11; myo-2p::mCherry]
raxEx73[rab-3p::nlp-11; myo-2p::mCherry]
raxEx74[rab-3p::nlp-11; myo-2p::mCherry]
raxIs119 [lbp-3p::lbp-3::gfp, myo-2p::mCherry];nlp-11(rax51)
raxIs86[lbp-8p::lbp-8::3xflag::sl2-RFP; myo-2p::GFP]
raxIs86[lbp-8p::lbp-8::3xflag::sl2-RFP; myo-2p::GFP]; raxIs141[lbp-3p::lbp-3::sl2-GFP]
raxIs132[lbp-2p::lbp-2::sl2gf; myo-2p::mCherry]
raxIs119 [lbp-3p::lbp-3::gfp, myo-2p::mCherry];
raxIs114[sur-5p:lmp-1::RFP-3XHA;unc-76(+)]
raxEx509[ges-1p::lbp-3::RFP::sl2-GFP]
raxIs3[ges-1p::lipl-4::sl2-GFP; myo-2p::mCherry];
nre-1(hd20); lin-15b(hd126)
nre-1(hd20); lin-15b(hd126)
raxIs119 [lbp-3p::lbp-3::gfp, myo-2p::mCherry];egl-21(n476)
Is[ges-1p::RDE-1::unc54 3'UTR, myo-2p::RFP3];rde-1 (ne219)
nhr-49(nr2014)
nhr-80(tm1011)
raxIs119 [lbp-3p::lbp-3::gfp, myo-2p::mCherry];nhr-49(nr2041)
raxIs3[ges-1p::lipl-4::sl2-GFP; myo-2p::mCherry];nhr-49(nr2041)
raxIs3[ges-1p::lipl-4::sl2-GFP; myo-2p::mCherry];nhr-80(tm1011)
rab-3p::nhr-49::unc-54 3'UTR, myo-3p::mCherry
raxIs119 [lbp-3p::lbp-3::gfp, myo-2p::mCherry];nhr-49(nr2041); rab-3p::nhr-49::unc-54 3'UTR, myo-3p::mCherry
raxIs3[ges-1p::lipl-4::sl2-GFP; myo-2p::mCherry]; fat-3(wa22);lbp-3(rax60)
raxEx586 [ges-1p::lbp-3(chim)::sl2::gfp]
raxEx587 [ges-1p::lbp-3(chim)::sl2::gfp]
raxEx581 [ges-1p::lbp-3::gfp]
raxEx599 [ges-1p::lbp-3(chim)::3xHA::sl2::gfp]
raxEx600 [ges-1p::lbp-3(chim)::3xHA::sl2::gfp]
raxEx602 [rab-3p::GBP::SAX-7::sl2::mKate2]
raxEx603 [rab-3p::GBP::sl2::mKate2]
raxEx604 [nhr-49p::nhr-49::mKate2];raxIs22[nlp-11::sl2gfp]
raxEx603 [rab-3p::GBP::sl2::mKate2];raxEx581 [ges-1p::lbp-3::gfp]
raxEx509[ges-1p::lbp-3::RFP::sl2GFP];raxIs3[ges-1p::lipl-4::SL2GFP]
raxEx548[egl-21p::egl-21::sl2::GFP]
raxEx609 [ges-1p::nslbp-3::RFP::sl2GFP]
raxIs3[ges-1p::lipl-4::SL2GFP];raxIs103[sur-5p:lmp-1::RFP-3XHA;unc-76(+)]
raxIs103[sur-5p:lmp-1::RFP-3XHA;unc-76(+)]
raxEx402[ges-1p::lipl-4::sl2-GFP];rde-1 (ne219); Is[ges-1p::RDE-1::unc54 3'UTR, myo2p::RFP3]

Wild animals

No wild animals were used in this study

Field-collected samples

No field-collected samples were used in this work

Ethics oversight

No ethical approval or guidance required

Note that full information on the approval of the study protocol must also be provided in the manuscript.
