# Supplementary figures and images for: Lysosome lipid signalling from the periphery to neurons regulates longevity
Source: Nat Cell Biol. 2022 Jun 9;24(6):906–16. doi: 10.1038/s41556-022-00926-8 (PMC9203275; doi:10.1038/s41556-022-00926-8)

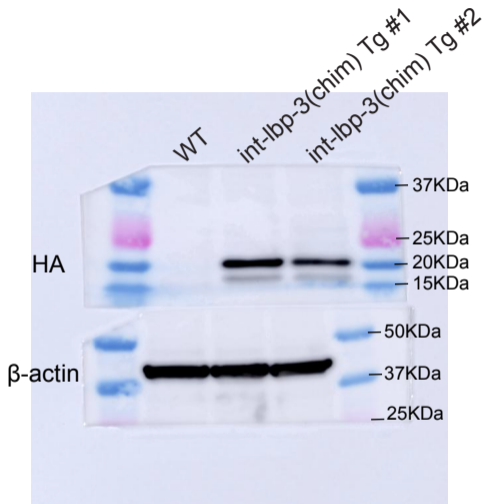

Supplement: Source Blot Extended Data Fig. 7 — Unprocessed western blot. [file 41556_2022_926_MOESM16_ESM.pdf]
